# Blends and Nanocomposite Biomaterials for Articular Cartilage Tissue Engineering

**DOI:** 10.3390/ma7075327

**Published:** 2014-07-22

**Authors:** Azadehsadat Hashemi Doulabi, Kibret Mequanint, Hadi Mohammadi

**Affiliations:** 1 School of Engineering, Faculty of Applied Science, University of British Columbia, Kelowna, BC V1V 1V7, Canada; E-Mail: hashemi.azadeh@gmail.com; 2 Department of Chemical and Biochemical Engineering, the University of Western Ontario, London, ON N6A 5B9, Canada; E-Mail: kmequani@uwo.ca; 3 Biomedical Engineering Graduate Program, the University of Western Ontario, London, ON N6A 5B9, Canada; 4 Biomedical Engineering Graduate Program, University of British Columbia, Vancouver, BC V6T 1Z4, Canada

**Keywords:** blend, nanocomposite, biomaterial, articular cartilage, tissue engineering

## Abstract

This review provides a comprehensive assessment on polymer blends and nanocomposite systems for articular cartilage tissue engineering applications. Classification of various types of blends including natural/natural, synthetic/synthetic systems, their combination and nanocomposite biomaterials are studied. Additionally, an inclusive study on their characteristics, cell responses ability to mimic tissue and regenerate damaged articular cartilage with respect to have functionality and composition needed for native tissue, are also provided.

## 1. Introduction

Polymeric materials are widely used in several biomedical fields. A large body of biomaterials have been considered and reviewed for tissue repairs [1,2,3]. There are a lot of literature surveys and comprehensive review papers covering several natural and synthetic polymers that have been studied for cartilage tissue engineering [4,5,6,7].

Among these polymers, natural polymers could provide some properties such as biocompatibility, biodegradability, low toxicity, and cell signaling [8,9] whereas synthetic polymers provide some other properties such as mechanical and physical properties and thermal stability [10]. Some of these polymeric three-dimensional (3D) scaffolds could provide microenvironments for cell supporting, cell migration, cell proliferation, cell fate and differentiation [11,12]. Not surprisingly, many of them do not provide sufficient mechanical properties to continuously support the formation of cartilage tissue. Some examples of widely used natural and synthetic biomaterials are summarized in Table 1.

**Table 1:** List of some natural and synthetic polymers have been extensively surveyed for cartilage tissue engineering.

Polymers	Examples
Natural polymers	Proteins: collagen [13], gelatin [14], fibrin glue [15] Polysaccarides: Agarose [16], alginate [17], cellulose [18], chitosan [19], chondroitin sulphate [20], and hyaluronic acid [21]
Synthetic polymers	poly(α-hydroxy esters): Poly(L-lactic-*co*-glycolic acid) [22], poly(ε-caprolactone) [23], Poly(NiPAAm) [24], poly(vinyl alcohol) [25], Polyurethane [26]

Synthetic polymers could be fabricated into different shapes with various microstructures [5] but the processability of natural polymers is more difficult than synthetic ones due to their sensitivity to process conditions such as temperature [27]. Although it is much easier to use synthetic polymers in the biomedical field, natural polymers are also required due to their specific characteristics mentioned above. Moreover, the pure polymer and also polymer blends could be strengthened by the inclusion of nanomaterilas [28]. Therefore, the use of materials that combine and provide the characteristic features of both natural and synthetic polymers is of interest.

As alluded above, the organized structure of natural polymers in comparison with synthetic polymers accounts the specific characteristics such as improved cell viability and tissue ingrowth [3] which is their single most advantage. In addition, some natural polymers (e.g., collagen and chondroitin sulphate) are present in cartilage tissues; therefore the similar structure could impart cartilage characteristics to the scaffolds [29].

Polymer blending, making composites, and synthesizing copolymers and interpenetrating polymer networks (IPNs) are some of different methods of polymer modifications and they are of significant interests, since these modifications could lead to the development of a new range of biomaterials with full set of desired properties [23,30,31,32]. The history of blending dates back to the in 19th century, when Parkes (1846) introduced the first blends of *trans*- and *cis*-1,4-polyisoprene including fillers [33]. Polymer blending and compositing of biomaterials have attracted an increasing attention over the last two decades due to biocompatibility and providing special properties considered for the application.

Polymer blends are mixtures containing two or more polymers and/or copolymers [33], while nanocomposites are comprised of polymers (natural; synthetic) and nanomaterilas, which refer to materials with a nano-sized topography or composed of nano-sized building components [27,34].

Since basic information on tissue engineering principles is amply available to the reader in the open literature, we focus on articular cartilage. Cartilage tissue engineering does not require extensive vascularization, hence a lot of polymeric scaffolds were evaluated [35] but clinical trials are limited and need to be assessed through long-term outcomes [36]. Synthetic polymers such as poly(lactic-*co*-glycolic) acid (PLGA) and poly(caprolactone) (PCL) are already used for clinically established products. It is worth to point out that some polymers, such as poly(urethanes) (PURs), poly(phosphazenes), and natural polymers such as collagen, fibrin and hyaluronic acid polymers are under current clinical investigation [37].

Articular cartilage is a connective tissue that lines the ends of articulating bones and provides frictionless motion in diarthrodial joints whilst protecting the bones of joints from being damaged when subjected to impact and load bearing. Unlike many other tissues, articular cartilage is the avascular, aneural, and alymphatic tissue so its ability to regenerate itself remains challenging [38]. Moreover, repairing articular cartilage in organized zones with functional mechanical properties such as viscoelasticity, anisotropy, nonlinearity and inhomogeneity has to be carefully considered and remains one of the primary obstacles in cartilage repair from a biomechanical standpoint. The purpose of this review is to highlight the available published information and recent progress on blends and biocomposite systems with respect to the preparation, evaluation, and their potentials as biomedical implant or scaffold materials for articular cartilage tissue engineering. The fabrication strategies for polymeric scaffolds are beyond the scope of this paper and will not be discussed here [6]. Firstly, the anatomy and properties of the natural articular cartilage tissue are discussed, and then the necessary requirements for engineering scaffolds are identified. Secondly, we address some of the most common natural and synthetic polymers including strategies for fabrication of blends and nanocomposite scaffolds. Finally, some of the most critical challenges for future approaches to form cartilaginous tissues that provide a functional replacement and restoration of articular cartilage from biomaterial point of view are introduced. Furthermore, a brief outline on the state of the art of current progress in terms of material properties, composite development and cartilage applications is presented.

## 2. Articular Cartilage Tissue Engineering

### 2.1. Structure-Property Relationships of Native Articular Cartilage

In order to engineer articular cartilage; there is a need to understand its composition; architecture and function for identifying the essential requirements for matrixes or scaffolds used for this application; because the composition and structure of extracellular matrix (ECM) within this tissue has a direct role in its function as a mechanical surface through regulation of its tensile, shear and compressive properties [39]. The primary roles of this highly specialized tissue are to facilitate articulation; distribute the loads uniformly to the underlying bone and provide the joint with essential biomechanical functions such as wear resistance; load bearing and shock absorption with low friction under high joint loads [40]. Articular cartilage is a connective tissue that lines the ends of articulating bones forming freely moving in diarthrodial joints [41]. It forms a layer; normally 3–4 mm thick and its whitish color is owing to its lack of vascularity [38]. The tissue is not only compositionally complex but also biologically active [42]. A growing body of literature investigating the cartilage tissue engineering has published papers about articular cartilage structure and biology in detail [39,43,44,45].

**Figure 1:**
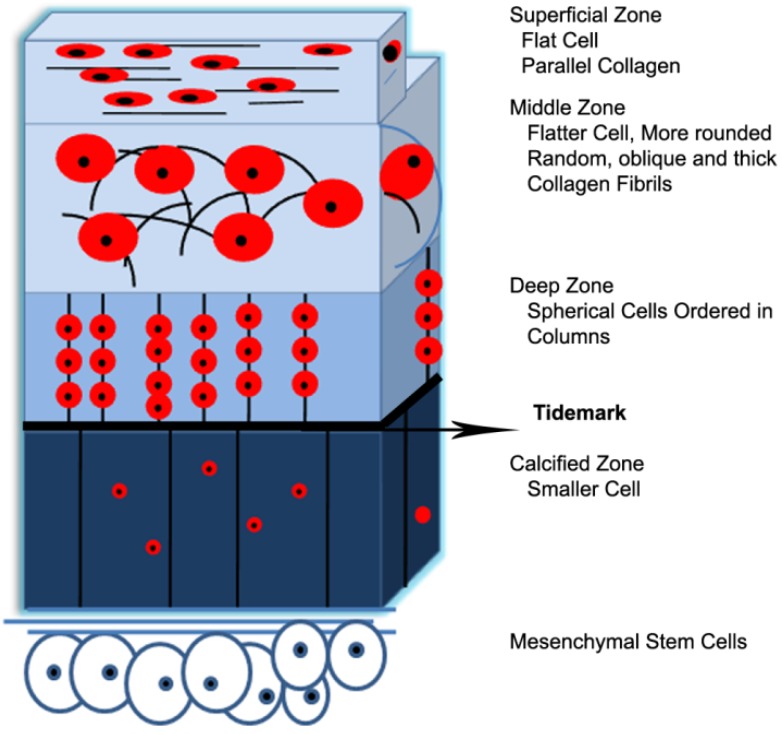
Zonal organization in normal articular cartilage, the black lines and the red solids represent collagen fibrils and chondrocytes, respectively.

Articular cartilage represents the only remnant of the original cartilage template to persist throughout adult life where it is divisible into four horizontal layers with their hierarchical network structure determining its mechanical behaviors [46,47]. The mechanics, structure, and organization of articular cartilage are only summarized here as it has been previously reviewed in detail by several authors [36,48,49]. With respect to depth within the tissue from the surface through to the bone [50], four distinct zones can be distinguished by extracellular matrix structure and composition, as well as cell shape and arrangement within the tissue [4,51] (Figure 1). In other words, the composition and organization of the matrix, cell morphology, orientation, density and metabolic activity, collagen fiber assembly and thickness vary from the surface respect to the depth and give it anisotropic properties in some mechanical environments [52]. In fact, these zonal gradients determined the functional and mechanical properties of articular cartilage; hence, designing and preparation of this tissue have remained challenging.

**Figure 2:**
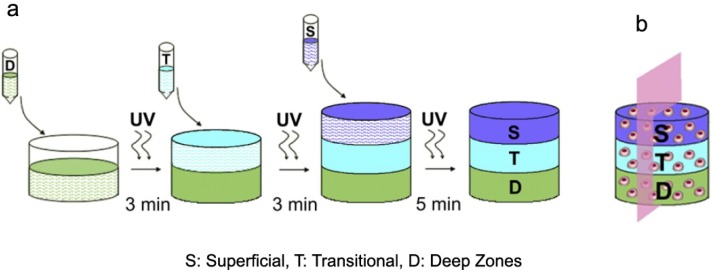
(**a**) Multi-layer scaffold fabrication with three distinctive layers; (**b**) Schematic of a cell laden in composite multi-layer hydrogel, reprinted with permission from [55]. Copyright 2011 Elsevier.

Three possible approaches were reported for articular cartilage repair: scaffold and matrix free, scaffold based and matrix based. According to zonal organization mimicking, matrix based approach is the effective one [53] in which scaffolds can be formed in various shapes and polymerized with different methods such as light curing, or with chemical materials and some of them could be injected into cartilage defect [54]. Nguyen *et al.* [55] fabricated 3D scaffolds with layer-by-layer organization of PEG-based hydrogel with chondroitin sulfate and matrix metalloproteinase-sensitive peptides, which were seeded with stem cells, resulted in to differentiate into zone-specific chondrocytes and organize into a complex tissue structure. The superficial zone composition consists of PEG, chondroitin sulfate and matrix metalloproteinase-sensitive peptides, the transitional zone composition consists of PEG, and chitosan, and finally the deep zone composition consists of PEG and hyaluronic acid [55] (Figure 2).

It is noteworthy that there is a second type of organization of articular cartilage at the microscale. At this classification, matrix structure and composition are varying with respect to distance from the chondrocyte membrane [50].

First zone, superficial zone, is characterized by having dense thin collagen fibrils parallel to the articular surface which possesses greater tensile stiffness and strength than the deeper zones, and it may resist shear forces generated during use of the joint as well as relatively low proteoglycan content, low permeability [56]. Enhancing the mechanical properties of the tissue surface is resulted from cross-linking of proteoglycans and collagens [56]. It has high-density flattened chondrocytes in ellipsoid-shaped but in small volume relative to the other cartilage zones. The main role of this zone is to facilitate the wear and provide low frictional surfaces of the tissue. Second zone is middle zone in which cells are spheroidal shape and appear randomly distributed in less parallel thick collagen fibrils. Proteoglycan content is at maximum level and the cell density is much lower than the superficial zone. This zone comprises 40%–60% of total thickness and several times the volume of the superficial zone [57]. Third zone is a deep zone in which the cells appear to line up vertically in short, oriented irregular columns in the direction of thick collagen fibrils perpendicular to the articular surface. The water content is lowest in respect to the other zones [58]. Its collagen structure is characterized by large fibers that form bundles oriented perpendicular to the articular surface. Proteoglycan content is much lower than in the middle zone, and the cell density is also the lowest of all cartilaginous zones [39]. The final zone is a calcified zone separated from the deep zone by the tidemark. The stiffness values vary between the more flexible cartilage and rigid bone. In this zone, the cells in small size are distributed randomly in a matrix filled with hydroxyapatitic crystals [58].

The functional properties of cartilage under compression (especially repeated loading) are highly dependent on fluid pressurization within the tissue [59]. The articular cartilage shows both creep and stress-relaxation behaviors [60,61]. When a compressive load is applied onto the tissue, fluid phase bears it, and the nonlinear permeability acts as a protective mechanism. Then the solid matrix begins to bear more of the load as the fluid exudes from cartilage. Finally by lasting time, just tissue bears the load [39]. It is worth to point out that articular cartilages show anisotropic behavior in some mechanical testing such as tension [62]. The nonlinear characteristics of articular cartilage are due to collagen recruitment and energy absorption properties of the tissue. These compressive properties are a direct result of its biphasic nature due to the nonlinear permeability response [63]. The viscoelastic properties of articular cartilage are dependent of the interactions between collagen and proteoglycan networks [60].

Modeling cartilage is challenging mainly because it becomes anisotropic when it undergoes deformation [64]. There are several theoretical models have been used for articular cartilage, such as physico-chemical, elastic and viscoelastic, biphasic/poroelastic, thermoanalog, electro-magnetic, biocompanent, fiber-reinforced, and triphasic models [48,65]. These models have several limitations. The physico-chemical model is based on Donnan theory applied for homogeneous only. The linear elastic model of articular cartilage cannot be implemented to model the time-dependent or dynamic behavior of the articular cartilage tissue such as the fluid flow or solid consolidation. Therefore, it may be only applicable for simplistic mechanical models of articular cartilage under static or quasi-static loading conditions [48]. Fiber-reinforced models are other ones, which are capable of capturing the material nonlinearity of the cartilage tissue but not the geometric nonlinearity [66] and the quantitative measurements of collagen orientation have been neglected. Under large deformation assumptions, linear elasticity is no longer valid and the solid phase behavior of the articular cartilage tissue is better represented by a viscous and hyperelastic material model [48]. Some researcher view several of the models of cartilage discussed as equivalent to treating articular cartilage as a porous rock with extensive flow of fluid, whereas, there is evidence that hydrogel models [67] better describe the physical behavior of articular cartilage, including being consistent with its low permeability in comparison with assessment performed by Maroudas *et al.* [68].

The most popular theory for articular cartilage compressive viscoelastic behaviors is the biphasic theory developed by Mow *et al.* [63]. This model considers the collagen-proteoglycan matrix as an elastic solid which is intrinsically incompressible, porous and permeable. The main advantage of this model is particularly efficient for addressing time-dependent aspect of cartilage mechanics [48]. Lai *et al.* [65] developed their theory in to triphasic model with considering electrolytes and ions as a separated phase. In other words, negatively charged proteoglycans are modeled to be fixed to the solid matrix, and monovalent ions in the interstitial are modeled as additional fluid phases. Swelling property of articular cartilage could be described by this model [65]. The thermodynamic plausibility of the triphasic theory was studied by Huyghe and coworkers. They reported that unphysiological generation of free energy during each closed cycle of loading and unloading conflicting with second law of thermodynamics was a limitation of the triphasic model [69]. The correspondence between equilibrium biphasic and triphasic material properties of articular cartilage was investigated by Ateshian *et al.* [70]. It is worthy of note; however, the triphasic model of cartilage provides a more accurate description of the tissue composition and mechano-electrochemical response, the biphasic model is preferable to use because of its ability to successfully describe the response of cartilage to various loading conditions [70].

Some mechanical properties of cartilage are summarized in Table 2. The data show that material selection and scaffold preparation, which fulfill these requirements, are very challenging subjects whereas the structure of zone organization should be mimicked [53,55]. In other words, the mechanical thresholds that engineered tissue will likely encounter after performance. From a biomechanical perspective, engineered scaffolds of articular cartilage should behave as a multiphasic fiber-reinforced permeable composite with inhomogeneous, anisotropic, nonlinear, and viscoelastic properties [71]. For example, it has been reported that the tensile modulus of human knee joint cartilages was higher in the superficial zone as compared to the other zones due to the different organizations and thickness variation of collagen fibrils within different zones [72]. Tensile and compressive moduli of native cartilage typically have two-orders of magnitude difference due to the tension-compression nonlinearity attributed to the reinforcing characteristics of the stiff collagen fibers embedded within the ECM [73]. Hence, tissue repair could be carried out using a liable approach to satisfy these zonal organization properties. There are some strategies to repair articular cartilage tissue with respect to the injury type.

**Table 2:** Mechanical properties of articular cartilage tissue (regardless of location).

Mechanical Properties	Articular Cartilage
Tensile Modulus (at 10% ε)	5–25 MPa [62,74]
Equilibrium Relaxation Modulus	6.5–45 MPa [63]
Elongation to Break	80% [32]
Ultimate Tensile Stress	15–35 MPa [75]
Equilibrium Compressive Aggregate Modulus ^a^	0.1–2.0 MPa [37]
Hydraulic Permeability	0.5–5.0 × 10^−15^ m^4^ N^−^^1^∙s^−^^1^ [37]
Intrinsic, Equilibrium Young’s Modulus in Compression ^b^	0.4–0.8 MPa [56]
Compressive Strength	14–59 MPa [76]
Equilibrium Shear Modulus	0.05–0.25 MPa [77]

^a^ It was calculated under confined compression mode [56]. ^b^ The intrinsic, equilibrium-Young’s modulus- of the solid matrix can be determined by the equilibrium response in the stress relaxation test. It was calculated under unconfined compression mode [56].

### 2.2. Joint Disease and Medical Interventions

There are different defects of articular cartilage with respect to the size, depth, and lesion locale including matrix disruption, partial-thickness and full-thickness [78]. Therefore, several treatments were suggested to repair and heal the defects [78]. Arthritis is one the most common disease caused trauma and inflammation hence cartilage repair is necessitate [79]. Exercises, use of anti-inflammatory medications and possibly an injection of steroid are some conservative treatments [78]. Tissue grafting, implantation of prostheses, autologous chondrocyte transplantation, microfracture, resection, and osteotomy are surgical interventions to repair cartilage [51,80,81]. Each treatment option has its own benefits, limitations, and clinical performance [37]. For example, donor site morbidity, risk of infection and inflammation of donor site, immune response and disease transmission are some important disadvantages of tissue grafting strategy. For example, donor site morbidity, risk of infection and inflammation of donor site, immune response and disease transmission are some important disadvantages of tissue grafting strategy [45].

### 2.3. Tissue Engineering of Cartilage and Scaffold Requirements

**Scheme 1:**
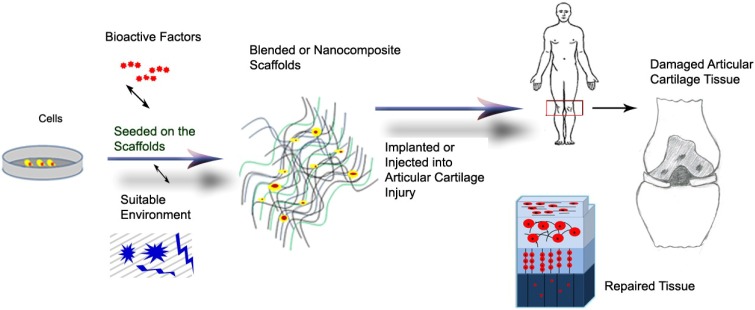
Schematic representation of the articular tissue engineering procedure.

Tissue engineering is a promising approach to repair or regenerate damaged tissues, organs with respect to recover them in the maximum efficiency with hopes of improving clinical outcomes. Tissue engineering could be a promising alternative approach to regenerate articular cartilage tissue using three main parts: cells, biomaterial scaffolds and environment including mechanical stimuli and bioactive factors (Scheme 1) [78,82]. Articular chondrocytes, stem cells (induced pluripotent cells, IPCs; mesenchymal stromal cells, MSCs; and human embryonic stem cells, hESCs) and their combination are some examples of cells used for tissue engineering [83]. Because of low cellularity, there is a limitation of cell sources to clinical translation. Stimulating environment factors (e.g., growth factors, oxygen tension, gene, drugs and bioreactors) used to encourage appropriate *in situ* cell differentiation, cell proliferation and chondrogenesis for cartilage tissue engineering on the scaffolds [45,81,84,85,86,87,88,89,90,91]. The emphasis of this paper is on biomaterial scaffolds.

There are some properties essential for scaffolds used as a matrix for articular tissue engineering to support cell fate and tissue mechanical properties for tissue or organ repair and regeneration [92,93,94,95]. The scaffolds should be porous and inter-connected so that cells could migrate and penetrate through the interstices. Surface properties of the scaffolds as well as materials play important roles such as cell attachment. Matrix biocompatibility and non-toxicity of degraded byproducts as well as matrix biodegradability are vital properties for good contact with native tissue compartment such as collagen fibrils and physiological remodeling, respectively. For enhancing interfacial integration between collagen fibrils and native tissue, the scaffold should be able to bond whilst it should be secured internal cohesiveness. There are some more factors, which should be considered for design and preparation of scaffolds, such as viscoelastic properties, which are responsible to resiliency during and following dynamic or static deformation; and structural anisotropy which provide anisotropic tissue organization similar to the native articular cartilage [45,84,85,86,89].

Scaffolds play a prominent role in encouraging chondrogenesis of the cell lineages due to providing structures for cells and facilitating the cellular and cell-matrix interactions, as well as affecting how cells sense mechanical loads [96]. They can be applied with cells or cell-free and also broadly categorized according to their chemical nature into natural, synthetic and their blend polymers. Composite and nanocomposite polymers are alternatives for engineered scaffolds of articular cartilage tissue [36]; they should help to mimic ECM structure and function of articular cartilage.

The success of tissue engineering depends on selecting materials with suitable properties such biocompatibility, cytotoxicity, suitable mechanical strength, implantability. Many natural polymers such as collagen [97], gelatin [98], hyaluronan [99], chondroitin sulphate [100], alginate [101], and chitosan [102] have been widely explored as promising biomaterials for tissue engineering, regenerative medicine and controlled delivery of biomolecules such as drugs, growth factors and cells to induce chondrogenic fate [89,103]. Although natural polymers are biocompatible, have good cell interactions and minimal stimulation to inflammatory or immunological responses of the host tissues they do not possess good mechanical properties. Modification of these polymers chemically or physically is an alternative process to improve their mechanical properties provided that their biological properties are not compromised [23,30,31].

## 3. Biomaterial Blends

Polymer blending is a well-known technique whenever property modification is required, because this inexpensive technology enables materials with full set of tailored properties and improved specific properties [104]. The main reason of blending is to widen the range of properties while obviating their drawbacks. Several advantages and disadvantages of some natural and synthetic polymers have been studied for the scaffold preparations of cartilage tissue engineering are summarized in Table 3. Usually the combination of natural polymers with synthetic polymers preserves the advantages of polymers. IPN or semi-IPN hydrogels are also other approaches to prepare materials that enable the scaffold mimics the structural properties of the articular tissue, as well as possess load-bearing properties [105].

**Table 3:** Advantages and disadvantages of several natural and synthetic polymers have been extensively studied for cartilage tissue engineering, reprinted with permission from [6]. Copyright 2010 Elsevier.

Polymers	Disadvantages	Advantages
Chitosan	Low tensile and compressive properties, low processability.	Antibacterial activity, low toxicity, good cell interaction, good biocompatibility, renewability, water solubility, stability to variations of pH.
Collagen	Low tensile and compressive properties, high degradation rate.	Low antigenicity, good cell adhesion, biological signaling, biodegradability.
Hyaluronic acid	Not support thermodynamically cell attachment. Hydrophilic surface.	No immunogenicity, good cell interaction.
Alginates	Hard processability, low tensile properties.	Injectable polymers, easily crosslinking under mild condition, high and tunable porosity scaffold, high diffusion rates of macromolecules, good cell incorporation.
Poly(ε-caprolactone)	Long term degradation application due to slow degradation rate, susceptible to undergo auto-catalyzed bulk hydrolysis, hydrophobic surface then no cell interaction.	FDA approval, easily processable.
Polyurethane	Acidic degradation byproducts in poly(esther urethanes) causing autocatalyzed degradation and *in vivo* inflammation.	Good tensile and compressive properties and also biological properties such as cell attachment, incorporation and supporting chondrocyte phenotype, and low infection.
PLGA	Low biological properties such as cell attachment, incorporation and supporting chondrocyte phenotype, releasing acidic degradation byproducts caused inflammatory response.	FDA approval, tailorable physicomechanical properties.

Blends of chitosan and collagen [106], collagen/hyaluronan/chitosan [107] and hydroxyapatite/collagen/chondroitin sulfate nanocomposite [108] are some examples of this classification. Sometimes, synthetic polymers, such as polyvinyl alcohol, are mixed with other synthetic ones to modify its drawbacks such as poor bioactivity. It must be borne in mind that the engineered articular cartilage should match the mechanical functionality of the native tissue. Therefore, engineered articular cartilages should have properties such as anisotropic, nonlinear, viscoelastic, and inhomogeneity. Preparation of composites based on material, which has a similar structure to glycosaminoglycans (GAGs) as a key component of cartilage ECM, could be considered as an attractive approach to achieve biomaterials for articular cartilage repair. Important characteristics of these polymer blends are provided in the following sections.

### 3.1. Blends with Collagen

Among natural biomaterials, collagen has attracted many interests because it is the most abundant protein constituting the natural ECM of articular cartilage which is responsible for expressing the chondrocytes phenotype, maintaining GAG production and supporting the chondrogenesis [84,109]. Collagen is synthesized by appropriate cells such as fibroblasts and osteoblasts [110,111]. The 90% of the dry weight of articular cartilage is composed of type II collagen in the form of crosslinked fibrils [112]. Collagen is a biocompatible, low antigenic and biodegradable polymer, which forms the extracellular framework, serves as a natural substrate for cell fate and provides the mechanical strength and shape of cartilages during articulation [97,111,113]. Adherent cells from bone marrow or periosteum were dispersed in collagen gel and transplanted into medial femoral condyle of rabbit model repair large and full thickness defect of articular cartilage [114]. It must be born in mind that the repair without chondrocyte transplantation resulted in fibrocartilage formation then the articular cartilage structure was not achieved [115]. The use of cells embedded in collagen gel instead of periosteal patches allowed rapid circumferential accumulation of newly synthesized matrix [116]; hence, the osteochondral progenitor cells had uniformly differentiated into chondrocytes and cartilage restored whilst mechanical properties of the repair defect were compliant [114].

**Figure 3:**
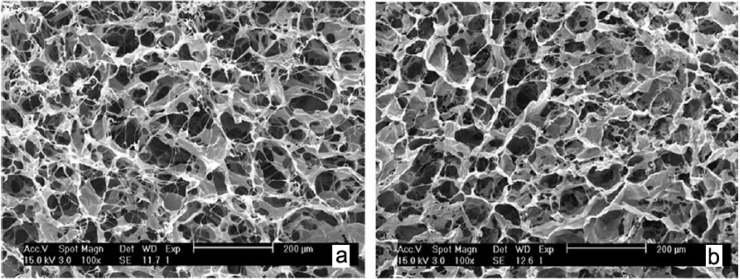
The SEM images of (**a**) uncross-linked and (**b**) 0.3% genipin cross-linked collagen/chitosan scaffolds with 90/10 in blend ratio, reprinted with permission from [106]. Copyright 2010 Wiley Periodicals, Inc.

Two important limitations of collagen are low mechanical stability and rapid biodegradation rate causing it not to be used alone for application of articular cartilage tissue engineering. Hence, crosslinking [117], making blend or composite crosslinked collagen with natural or synthetic polymers are important approaches to overcome these problems [118]. Blend scaffolds of crosslinked collagen and chitosan are reported to have higher mechanical stability than pure collagen [106]. In this cited study, cell attachment and proliferation and viability were evaluated and revealed that the blended scaffolds were biocompatible and noncytotoxic and it may serve as a 3D matrix for cartilage tissue engineering [106]. As chitosan has more amino groups than collagen more cross-linking points will lead to higher biostability. Since collagen and chitosan are miscible at molecular level, they exhibit hydrogen bonding or electrostatic interaction that could reinforce the mechanical stability [106,119]. Adding chitosan to collagen could increase these susceptible sites to be crosslinked therefore it is difficult for enzymes to access the cleavage sites in collagen thereby increasing biostability and easily regulate the degradation rate (Figure 3) [106,118].

Lin *et al.* [107] also indicated that collagen/chitosan/hyaluronan hybrid polymer scaffold showed great potential as a desirable biomaterial for articular cartilage due to its excellent characters of cell adhesion, proliferation and matrix secretion. As chitosan shows cationic behavior due to its glucosamine residues and hyaluronan is an anion polysaccharide, tight ionic interaction bonds are present among them. The existence of such interactions of all compartment molecules could influence mechanical properties [107]. This behavior has been reported for collagen/chondroitin sulphate/chitosan hybrids in comparison to collagen scaffolds [29]. The scaffolds of collagen/chondroitin sulphate were not strong enough but incorporation of chitosan resulted in importing ionic interaction, tensile strength, and Young’s modulus [29]. Moreover, collagen/PVA nanofibers scaffold seeded with autologous MSCs was prepared by Abedi *et al.* [120] for the osteochondral defects repair of the rabbit joints due to the possibility of controlling cell fate to chondrogenicity.

**Figure 4:**
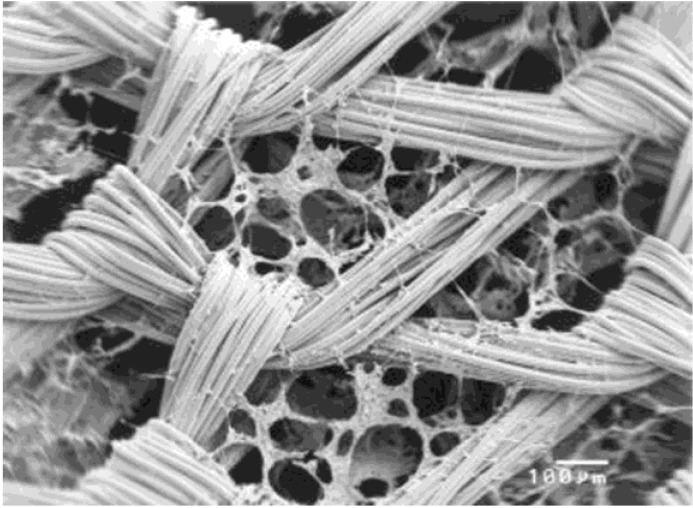
The SEM images of chondrocytes on the PLGA/collagen web after 1 day culturing, reprinted with permission from [121]. Copyright 2003 Wiley Periodicals, Inc.

Biochemical and architectural development of the blend scaffolds could be regulated by chemical and mechanical environments. For example, cell differentiation and ECM production could be influenced by surface area, pore size and interconnectivity of the scaffold mesh. Chen *et al.* [121] prepared web-like collagen microsponges in the knitted mesh of PLGA with adjustable thickness and enhanced biomechanical properties (Figure 4). Overall, MSCs cultured in PLGA/collagen cobweb-like scaffold for ten weeks showed the mentioned microenvironment could induce chondrogenic differentiation due to the specific tailored architecture of the composite mesh. Switching gene expression from type I collagen to type II collagen, cell density and distribution uniformity throughout the scaffold as well as comparable mechanical properties to native articular cartilage indicated that this scaffold is applicable for articular cartilage repair [122].

### 3.2. Blends with Chondroitin Sulphate

Chondroitin sulphate is of GAGs, which is a major component of the ECM of cartilage [123]. ECM molecules of cartilage could regulate metabolism and gene expression in chondrocytes as well as stimulate cell proliferation and differentiation [124]. It was reported that chondroitin sulphate presence could influence mechanical properties of the scaffold, demonstrating its incorporation with desired guest materials could enhance compressive strength due to proteoglycan secretion promotion [125]. Nanda *et al.* [124] fabricated a hydrogel scaffold of PVA/chondroitin sulphate imitating the ECM thereby provides suitable environment for better articular cartilage repair *in vivo*. They studied the effect of hydrogel composition and crosslinking agent content on the physico-chemical, and mechanical properties of the scaffold. As a result, the surface of the hydrogel not only could fill the cartilage defect without any inflammation but also could integrate with surrounded tissues. Therefore, chondroitin sulphate release from the scaffold due to biodegradability could play an important role in articular cartilage repair for regulate metabolism. In a different approach, Ko and coworkers [126] attempted to mimic the native ECM of articular cartilage by incorporating type II collagen with chondroitin sulphate and hyaluronan to up-regulate biosynthetic activities. This group used freeze-drying and chemical crosslinking procedures to fabricate highly porous and interconnected network composite scaffold. Since type II collagen provides mechanical properties such as tensile strength which influences the bioactivity of the seeded chondrocytes and performs important biological functions, its incorporation of type II collagen with chondroitin sulphate could show synergistic effect on cartilage repair [125]. Not only material properties and scaffold pore structure could influence cell fate but also applying mechanical forces such as pressure gradient, fluid flow, mechanical strain can support and regulate chondrogenic lineage of MSCs [127]. Chen *et al.* [128] fabricated a microenvironment of composite based on chitosan, chondroitin sulphate and dermatan sulphate, which plays critical roles in stimulating ECM production and cartilage regeneration in the native articular cartilage ECM for mimicking native tissues. They used Response Surface Method to predict the incorporation of GAGs with chitosan then the optimal formulation with considering proper amounts and ratios was predicted [128]. In line with this, incorporation of gelatin, chondroitin sulphate with hyaluronan in fibrin glue, which makes a suitable environment for cell distribution and seeding was assessed by Chou *et al.* [129].

### 3.3. Blends with Chitosan

Chitosan, a deacetylated derivative of chitin, is the linear polysaccharide composed of glucosamine and *N*-acetyl glucosamine linked in a β(1-4) manner with cationic nature and high charge density in solution [130]. Due to its beneficial intrinsic properties such as biodegradablity, biocompatiblity, nonantigenicity, nontoxicity, biofunctionality, antimicrobiality besides similar characteristics with various GAGs and hyaluronic acid present in articular cartilage, it is well known functional aid in terms of connective tissue in-growth and neo-vascularization for the ordered regeneration of human cartilage tissues [9,131]. Although chitosan has a lot of intrinsic properties, which make it applicable for tissue engineering applications, but chitosan suffers from a relatively poor mechanical characteristic, therefore preparing blends and composites with other biomaterials attracted much interest [132,133]. Incorporation of chitosan into another biopolymer, such as collagen and alginateimproved its mechanical property and reduced the biodegradation rate [134]. In order to improve cell attachment, cell proliferation and biosynthetic production as well as spherical morphology maintenance, blending and surface modification with chitosan are two important approaches [135]. Alginate, a family of polyanionic copolymers derived from brown sea algae, could promote cell proliferation, maintain cell functionality and enhance phenotype [136]. Li *et al.* [135] fabricated chitosan/alginate scaffold via thermally induced phase separation followed by freeze drying. Combining chitosan with alginate led to increased cell viability, promoted production of type II collagen as well as retaining spherical morphology; therefore, these scaffolds could be used for cartilage repair. A new approach to prevent osteoarthritis development was attempted by Oprenyeszk *et al.* [133] who prepared a biphasic biomaterial scaffold comprised of alginate-chitosan beads dispersed in chitosan hydrogel and injected into rabbit knee. The overall outcome showed the presence of alginate/chitosan beads could affect cartilage structure and cellularity, maintain the morphology of chondrocytes, and decreased friction coefficient [133].

Poly(L-lactide*-co*-ε-caprolactone) (PCLC), a highly viscoelastic biomaterial with hydrophilic nature could be used to simulate the elastic collagen in the ECM of articular cartilage tissue when applied *in vitro* for long-term cyclic compression on the cell–scaffold construct [137]. Yan *et al.* [138] produced and characterized PCLC scaffold modified with chitosan and seeded with human mesenchymal stem cells (hMSCs) for achieving appropriate surface properties such as wettability and ionic charges essential for cell attachment. The porosity was kept constant while improving cell functionality and induction differentiation. Overall outcomes revealed the GAGs deposition, type II collagen production (ECM protein expression) as well as cartilage tissue formation through PCLC modified with chitosan were higher than PCLC scaffold due to improvement cell distribution through the scaffold. This was probably due to overcoming on confined high aggregation of cells within a small region. Furthermore mechanical properties such as elasticity improved relatively to PCLC alone and deformation recovery ratio was very similar to native tissue. The confined high aggregation of cells within a small region in the PCLC scaffold might have caused a deficit in nutrient supply locally, thus impeding long-term cellular proliferation [138]. The final Young’s modulus of modified scaffold was lower than the native articular cartilage hence some further research should be performed to overcome this inefficiency.

### 3.4. Blends with PVA

Given PVA’s anisotropy, viscoelastic and biphasic nature, high permeability to fluid, and biotribological properties, PVA hydrogels have been extensively studied and employed in artificial articular cartilage repair [63,105].

Fabrication of PVA hydrogels via freeze-thaw process makes them to exhibits mechanical properties approximately similar to articular cartilage [139]. Moreover, it seems that the PVA plays an effective role for chondrocyte activity [140]. In view of this, its clinical applications are still limited due to limited stability for long-term utility, and non-bioactivity *in vivo* in comparison with native cartilage [31]. There are some techniques to modify PVA, for instance, co-polymerization, blending, or compositing with nanoscale materials [31,141]. Such modification may influence biological properties such as biocompatibility or cytocompatibility of composites hence selecting suitable second material could be challenging [142].

However PVA was a candidate material for articular cartilage repair, preparation PVA with acrylamide as a crosslinking agent was one of strategies used to increase PVA water permeability thereby improving the lubrication for articulation [31,143]. Adding hydrophilic polymers such as polyethylene glycol or acrylamide while preparation could be an approach to increase lubricity as well as stability by preventing pore collapses due to removing from the hydrogels.

The presence of hydrogen bonding between hydroxyl groups of polymer surface and polar groups on cell surface makes PVA to be very adhesive for cells but this property should be tailored. PCL with hydrophobicity has been well known for cell attachment therefore this property would be controlled by blending these polymers together to balance hydrophilic-hydrophobic moieties. Mohan and Nair [144] have prepared a porous scaffold of PVA/PCL followed modifying with Arginine-Glycine-Aspartic acid (RGD) peptide coupling to impart cell anchoring and stable linking for chondrocyte attachment. They reported that PCL acted as reinforcement for PVA in the semi IPN scaffold with viscoelastic characteristics, provided a favorable cell environment for GAG secretion as well as good medium uptake ability; hence the supply of nutrients to cells penetrated within the scaffold would be adequate [144]. Similarly, the effect of some signaling molecules released from the scaffold like TGF-β1, TGF-β3, and BMP-2on the MSC fate and secretion of ECM molecules was also evaluated [145]. It was shown that the scaffold microenvironment promoted better differentiation of MSCs to chondrocytes.

Preparation of blends based on biomaterials with similar structure to ECM or with similar mechanical properties to hyaline cartilage could be appropriate strategy for articular cartilage repair. Lee *et al.* [146] used porous scaffolds of PVA and *N,O*-carboxymethylated chitosan (NOCC) to evaluate mechanical properties for articular cartilage tissue engineering applications. NOCC induces suitable results in regard to articular cartilage, because it is a derivative of chitosan, a semi-natural polymer similar to GAGs and may play role in modulating the morphology, differentiation, and function of chondrocytes. In line with this, Ibrahim *et al.* [147] proposed the use of hydroxyapatite reinforced bilayered scaffolds of PVA/NOCC seeded with hMSCs as a promising biomaterial to repair osteochondral defect. Since hydroxyapatite is osteoconductive, the first layer could mimic bone and increase osteocyte proliferation structure [148] while the mechanical properties of the scaffold’s upper layer mimics articular cartilage [146]; therefore; these biocompatible bilayered scaffolds would be promising candidates for osteochondral damage [147].

Tribiology and wear characteristic of PVA as an articular cartilage replacement as well as wear mechanism in reciprocating sliding against articular cartilage under certain conditions was assessed by Sardinha *et al.* [149]. These authors demonstrated that very low friction coefficient values from 0.02 to 0.05 and no degenerative changes with different applied loads or lubricant fluid were obtained for PVA. To increase water absorption of PVA hydrogel, Polyvinylpyrrolidone (PVP), is a good alternative material for blending due to amide groups with strong hydrophilicity rather than hydroxyl and carboxyl groups. Enhanced water content value up to 1.14% for 15 wt% PVP due to PVP addition causes friction coefficient decrement needed for hyaline cartilage during articulation, as well as providing viscoelastic behavior similar to articular cartilage tissue [150]. Ma *et al.* [151] evaluated some parameters on the tribiology behavior of PVA/PVP blends. The presence of bovine serum as a lubrication fluid in the blended material showed minimal wear behavior (friction coefficient: *ca.* 0.05) due to the formation a thick fluid film for friction reduction.

Long fixation of scaffolds comprised of PVA could be achieved by preparation of hydrogel scaffolds contain inter-connected porous network. Scholten *et al.* [152] used alginate microspheres for fabrication IPN scaffolds of PVA to support chondrocyte phenotype. The authors demonstrated that the presence of internal porous network within hydrogel not only could facilitate integration with the host tissue as well as promoting inward cellular migration but also the blended scaffold could perfectly control load bearing behavior similar to that of articular cartilage after implantation [152]. In another study, Bichara *et al.* [153] fabricated neocartilage tissue from PVA/alginate hydrogel cultured with human nasoseptal chondrocytes, demonstrating auricular cartilage achieved by a predefined-shape-specific construct via controlling gelation kinetics.

## 4. Biomaterial Nanocomposites

Articular cartilage tissue shows unique combinations of nonlinear properties as well as anisotropic behaviors due to composition and orientation of tissue components [63]. Scientists have been interested in regenerating articular cartilage using nanobiomaterials to achieve similar mechanical and physical properties with the natural tissue [154]. Nanocomposite refers to multiphase materials at the nanometric scale within polymer matrix could mimic native tissue properties; therefore, it could be another approach to gain this goal. These nanobiomaterials in different shapes, such as particles, fibers, *etc.*, are mixed at the nanometer scale and they could show specific characteristics respect to the considered properties [155].

### 4.1. Polymer-Polymer Nanofiber Composites

It has been reported that ECM mimicry and maintenance of the chondrocytic phenotype are enhanced or promoted on the nanofiber scaffolds [156]. Li *et al.* [85] showed the enhanced chondrogenesis on nanofibrous scaffolds of the PCL in comparison with microfiber ones. Furthermore, Casper *et al.* [92] fabricated chitosan-coated PCL nanofiber scaffolds and placed them in the subperiosteal environment that behaves as a bioreactor and, hence, periosteal cells would penetrate into the scaffolds. They have reported that coating does not support cell penetration, proliferation and chondrogenic differentiation while this notion is in contrast to several published reports [102]. Likewise, Wise *et al.* [157] have reported that oriented nanofiber scaffolds of PCL could mimic the cell and ECM organization presented in the superfacial zone of articular cartilage. In another research, Coburn *et al.* [156] have demonstrated the chondrogenesity of nanofiber composite-base of PVA-methacrylate and chondroitin sulfate-methacrylate for articular cartilage repair. The low density scaffolds were cultured with MSCs for six weeks in both chondrogenic induction medium and *in vivo*. The presence of PVA with non-adhesive nature not only resulted in fibroblast invasion reduction *in vivo* but also enhanced GAG production while the presence of chondroitin sulfate in the fibers had a positive impact on increasing type II collagen synthesis and mechanical properties of tissues. Cell proliferation and differentiated into the chondrogenic lineage were confirmed by producing patterned ECM features and cartilage specific gene expression due to early cell infiltration and cartilage repair in an *in vivo* osteochondral defect of rat model [156]. Shin *et al.* [158] developed nanofiber scaffold of blend of PLGA (a blend of PLGA_75/25_ and PLGA_50/50_ in 1:1 blend ratio; 75/25 and 50/50 are lactic acid/glycolic acid ratios) and cultured them with porcine articular cartilage cells. Cellular responses such as GAG content, DNA content, as well as physical and mechanical properties, showed these blends were appropriate candidates for articular cartilage regeneration [158].

### 4.2. Polymer-Silica Nanoparticle Composites

Polymer-silica composites are another attractive class of materials for cartilage regeneration [159]. For example, Buchtova *et al.* [160] have fabricated improved hydrogel based on siloxane derived hydroxypropylmethylcellulose with mesoporous silica nanofibers, and yield efficient bionanocomposite ECMs resulted in mimicking cartilage tissue. The nanocomposite possessed dispersion at the nanoscale due to the chemical affinity between the hydrophilic silica nanofibers and the pendant silanolate groups of the polymer chains influenced gel point. Tuning the amount of nanocharges resulted by adding small amount of anisotropic, rod-like shape of the silica nanofibers, mechanical properties of the nanocomposite supported cartilage tissue requirements. Despite this, low compressive elastic moduli in comparison with native tissue caused them not to be applicable for articular cartilage repair except as a favorable environment for cells [160].

The feasibility of PVA/Si nanocomposite fabricated from PVA and tetra ethoxy silanena nospheres by sol-gel method for articular cartilage has been studied [80]. This nanocomposite could improve adhesion of the hydrogel on underlying bone with high bond strength so that it could not be loosen their integrities under applied loads [80]. Although these results show the improvement of durability of hydrogel and adhesion to the underlying tissue, further study is required in order to mimic composition and zonal organization of articular cartilage.

Enhancement of hydrogel mechanical properties, control and sustained *co*-delivery of biomacromolecules and therapeutic agents, as well as induce chondrogenicity function, are achieved by using mesoporous structure of silica nanoparticles. Zhu *et al.* [28] fabricated controlled release systems of composite hydrogel based on chitosan and silica nanoparticle for drug delivery applications. Introduction of nanoparticles with the rigid structure in hydrogels and then the existence of strong intermolecular interaction between nanoparticles silanol groups as well as chitosan susceptible groups resulted in improving hydrogel strength. Chitosan could use as a drug delivery system for biomacromolecules due to its positive charges but studies demonstrated a difficulty for hydrophilic drugs [161]. Introducing silica nanoparticle with mesoporous structure could resolve the release problem as well as produced GAGs and DNA demonstrate chondrocyte fate promotion due to lower concentration of released silicate ions in the inner environment [28].

### 4.3. Polymer-Hydroxyapatite Nanoparticle Composites

Hydroxyapatite nanocomposites have been used in several application fields especially in bone tissue engineering [148]. Hydroxyapatite is a biocompatible and bioactive material for construction of bone composition and it is osteoconductive [162]. Many studies demonstrated polymer-hydroxyapatite nanocomposites can stimuli osteoblast growth and proliferate [163]; however, there are also some studies regarding to cartilage tissue engineering and cartilage replacement [164,165]. The incorporation of these nanopoarticles with polymers, such as PVA, PLLA and chitosan can obviate some drawbacks of pure hydrogels by enhancing its physical and mechanical properties, biocompatibility, bioactivity and elasticity [165,166,167].

The use of bilayered or biphasic scaffold structures is an approach to promote the regeneration of articular cartilage while allowing for the repair of the underlying subchondral bone [168,169]. 3D macroporous chitosan-hydroxyapatite bilayered scaffolds were synthesized by Oliveira *et al.* [167] via a two-step procedure including sintering and freeze-drying as a bone layer and a cartilage layer, respectively. When bone marrow stromal cells (GBMCs) were cultured on these materials showed that the spongy structure with anisotropic porosity, adequate pore size and distribution with interconnectivity were supportive structure for cells to differentiate GBMCs into the chondrogenic lineage. Fabrication of controlled design of such scaffolds play more important role from manipulation point of view and make them good promising tissue substitutes for the regeneration of osteochondral defects but it may not be adequately applicable for the repair of articular cartilage of large animal models [170].

Shi *et al.* [171] prepared porous scaffolds of nanocomposites based on amphiphilic chitosan and hydroxyapatite by freeze-drying method for tracheal cartilage regeneration application. These scaffolds with pore size in the range of 200–500 μm and porosity more than 85% were cultured by rabbit chondrocytes and implanted subcutaneously into nude mice for eight weeks. The results of histomorphologic and biochemical assays revealed neonatal cartilage-like tissue were generated which were similar to mature hyaline cartilage.

As mentioned previously, PVA is a high promising material for cartilage [139] but its durability and week tissue adherent for a long term may not be ignored [172]. It has been claimed that compositing PVA with a bioactive and biocompatible material such as hydroxyapatite could improve fixation ability of the scaffolds to the surrounded tissues [173,174]. Pan and Xiong [166] have reported that the combination of PVA with hydroxyapatite can enhance not only mechanical properties and bioactivity but also adherent to around natural tissue [175]; therefore the aforementioned problem of PVA was omitted. They prepared PVA/hydroxyapatite nanocomposite by *in situ* synthesis method and incorporation with several repeated freeze-thaw cycle process (frozen at −20 °C for 12 h, and then thawed at 25 °C for 6 h). Viscoelastic properties of these PVA/hydroxyapatite nanocomposites were similar to viscoelastic behavior of articular cartilage [176]. Qu *et al.* [169] studied on PVA/gelatin/nano hydroxyapatite/polyamide-6 bilayered scaffolds via freeze-drying method for *in situ* osteochondral defect repair. The highly porous scaffolds possessed inter-connective pores structure assures morphological features necessary for regenerative medicine. Since gelatin may facilitate cellular adhesion and proliferation [31] and also PVA showed a good integrity and functionality of the osteochondral construct; hence, nanohydroxyapatite/polyamide-6 serves as a skeleton, providing the composite with high mechanical strength and promotes cell adhesion and proliferation. These scaffolds possessed biphasic behavior, which showed articular cartilage behavior in the upper layer and bone behavior within the lower layer [169]. The biocompatibility and chondrogenesis of the PVA- nano hydroxyapatite/polyamide-6 bilayered scaffolds were studied by culturing neonatal rabbit MSCs for two and results demonstrated good influence on the growth, proliferation and differentiation of chondrogenic MSCs. *In vivo* results revealed the induced BMSCs after seeding were well distributed in PVA-nano hydroxyapatite/polyamide-6 scaffold, in which they produced sufficient ECM to chondroid and or osteoid aggregates, and the neo-cartilage formed a new matrix, which was not seen in other bilayered scaffolds [177].

Polylactide (PLA) is of biocompatible, biodegradable and renewable materials, which could be modified to enhance its biological properties for cartilage engineering applications [178]. Spadaccio *et al.* [165] prepared a composite of electrospun PLA nanofiber with nanoparticle-hydroxyapatite to obviate the mentioned drawback as well as mimic the native histoarchitecture of osteochondral tissue thereby inducing chondrogenic differentiation of hMSCs toward chondrocyte by providing biological signaling and the biochemical effect of hydroxyapatite. It is worthy of note that the presence of hydroxyapatite may balance the pH of the cell environment containing acidic degradation byproducts. Tamai *et al.* [30] developed a triple composite scaffold of nanohydroxyapatite and poly-D,L-lactic acid/polyethylene glycol (PLA-PEG) as a carrier for recombinant human bone morphogenetic protein-2 (rhBMP-2) to stimulate the *in vitro* and *in vivo.* However, the mechanical properties of the scaffolds have not been reported; however, this bioactive scaffold could promote the repair of full-thickness articular cartilage defects within three weeks and hyaline cartilage appearance with a columnar organization of cells and mature matrix was observed after six weeks [30].

## 5. Conclusions

There is an increasing need for novel materials with desired properties for tissue engineering and regenerative medicine. This review article outlined the significant studies that have been performed in cartilage tissue engineering especially articular cartilage. Recent progress in the preparation material blends (natural-based and synthetic-based) and nanocomposite, as well as fabrication porous scaffolds with tailored properties, in the field of articular cartilage tissue engineering have been discussed. Having advanced engineering approaches suggests combination of material and biological science as well as technology needed for preparation novel biomaterials and for designing and controlling the polymeric scaffolds for tissue repair as the synergistic combinations of material characteristics and scaffold design play critical roles in cell interaction, provide mechanical properties and then tissue regeneration. A lot of research papers published regarding blends and composite materials for tissue engineering reveal there is a huge interest in this field; however most of results are far from clinical trials. In the near future, one can design multilayered biomaterial scaffolds, which may adequately mimic human articular cartilage tissue with respect to property and functionality of each zone while provides functional performance needed in long-term use with no sign of inflammation *in vivo*.
